# Dynamic liquid biopsy components as predictive and prognostic biomarkers in colorectal cancer

**DOI:** 10.1186/s13046-022-02318-0

**Published:** 2022-03-15

**Authors:** Afsheen Raza, Abdul Q. Khan, Varghese Philipose Inchakalody, Sarra Mestiri, Zeenath Safira K. M. Yoosuf, Takwa Bedhiafi, Dina Moustafa Abo El-Ella, Nassiba Taib, Shereena Hydrose, Shayista Akbar, Queenie Fernandes, Lobna Al-Zaidan, Roopesh Krishnankutty, Maysaloun Merhi, Shahab Uddin, Said Dermime

**Affiliations:** 1 Translational Cancer Research Facility, National Center for Cancer Care and Research, Hamad Medical Corporation, Doha, Qatar; 2 Translational Research Institute, Academic Health System, Hamad Medical Corporation, Doha, Qatar; 3 College of Health and Life Sciences, Hamad Bin Khalifa University, Doha, Qatar; 4 College of Medicine, Qatar University, Doha, Qatar; 5 Translational Research Institute and Dermatology Institute, Academic Health System, Hamad Medical Corporation, Doha, Qatar

**Keywords:** Liquid biopsy, Colorectal cancer, Microbiome, Exosomes, Circulating tumor DNA, Circulating noncoding RNAs, Circulating tumor cells

## Abstract

Colorectal cancer (CRC) is one of the most common cancers worldwide. The diagnosis, prognosis and therapeutic monitoring of CRC depends largely on tissue biopsy. However, due to tumor heterogeneity and limitations such as invasiveness, high cost and limited applicability in longitudinal monitoring, liquid biopsy has gathered immense attention in CRC. Liquid biopsy has several advantages over tissue biopsy including ease of sampling, effective monitoring, and longitudinal assessment of treatment dynamics. Furthermore, the importance of liquid biopsy is signified by approval of several liquid biopsy assays by regulatory bodies indicating the powerful approach of liquid biopsy for comprehensive CRC screening, diagnostic and prognostics. Several liquid biopsy biomarkers such as novel components of the microbiome, non-coding RNAs, extracellular vesicles and circulating tumor DNA are extensively being researched for their role in CRC management. Majority of these components have shown promising results on their clinical application in CRC including early detection, observe tumor heterogeneity for treatment and response, prediction of metastases and relapse and detection of minimal residual disease. Therefore, in this review, we aim to provide updated information on various novel liquid biopsy markers such as a) oral microbiota related bacterial network b) gut microbiome-associated serum metabolites c) PIWI-interacting RNAs (piRNAs), microRNA(miRNAs), Long non-coding RNAs (lncRNAs), circular RNAs (circRNAs) and d) circulating tumor DNAs (ctDNA) and circulating tumor cells (CTC) for their role in disease diagnosis, prognosis, treatment monitoring and their applicability for personalized management of CRC.

## Background

Cancer is a leading cause of death worldwide with an estimated 19.3 million new cancer cases and 10.0 million cancer deaths reported in 2020 [1]. In 2019, World Health Organization (WHO) reported that out of 183 countries, cancer is the first or second leading cause of death in 112 countries while it ranks third or fourth in further 23 countries [2].

Colorectal cancer (CRC) is the second leading cause of cancer-related deaths worldwide (9.4%) and ranks third (10%) in terms of newly diagnosed cases [1]. The diagnosis of CRC is mainly based on invasive colonoscopy computed tomographic (CT) colonography, computed tomographic colonography (CTC) or flexible sigmoidoscopy for biopsy of suspicious lesions. In addition to these, non-invasive diagnostic screening stool tests including fecal immunochemical test (FIT), guaiac-based fecal occult blood test (gFOBT) and multitargeted stool DNA test (Cologuard) are utilized frequently. Cologuard is an FDA approved screening test that targets methylated N-myc downregulated gene 4 (NDRG4), Bone morphogenetic protein 3 (BMP3), Kirsten rat sarcoma virus (KRAS) mutations and hemoglobin in stool samples. It exhibits specificity of 86.7% and sensitivities of 92.3 and 42.4% for detecting stage I–IV stage CRC and advanced adenomas (AA). On the other hand, methylated septin 9 (mSEPT9) (Epi proColon) is an FDA approved kit for the detection of DNA methylation marker, SEPT9, in circulating tumor DNA (ctDNA) in serum. Though both kits target DNA methylation markers for screening, their target genes differ possibly because tumor DNA from stool originates directly from gut while ctDNA from serum passes through various barriers of the body [3]. Furthermore, for treatment monitoring serum tumor markers such as carcinoembryonic antigen (CEA) and carbohydrate antigen 19–9 (CA19-9) are also commonly used [4, 5]. Although, all these tools are considered beneficial, there is growing body of evidence that reports limitations associated with these modalities. For e.g. complexities in colonoscopy include invasiveness, extensive patient preparation, risk of bowel tears/bleeding and high cost [5]. On the other hand, tissue biopsy extracted through colonoscopy is also associated with several technical complexities such as single tumor location picture, limited assessment of spatial tumor genetic heterogeneity, insufficient specimen, difficulty in longitudinal monitoring and requirement of well-trained personnel for testing [6, 7]. Similarly, non-invasive stool and tumor marker testing reports low sensitivities/specificities and may mostly require confirmation by colonoscopy [5]. Since CRC is a preventable and curable disease when diagnosed early, it is imperative that non-invasive tools with high specificity and sensitivity are identified to help in its early detection, prognosis, and treatment monitoring.

Cancer cells are known to have rapid turnover rates and as such release cancer associated cell products such as circulating tumor cells (CTCs), cell-free circulating nucleic acids (Cf DNA/RNA), microRNAs (miRNAs), long non-coding RNAs (lncRNAs), exosomes and proteins from primary or metastatic tumor into the extracellular environment. These biomarkers are released directly or indirectly into body fluids such as peripheral blood, urine, saliva, ascitic fluid, pleural effusion, cerebrospinal fluid and are known to provide important information on physiological processes at single cell level [8]. The analysis of extracellularly released biomarkers in body fluids is known as “liquid biopsy” and its importance in cancer screening, patient stratification, and monitoring has been documented extensively. In CRC, the importance of liquid biopsy is particularly stressed upon as this is a highly heterogeneous tumor with molecular characterization necessary for effective monitoring and management. In lieu of this, several liquid biopsy tests (for research use/FDA/IVD approved) are currently in use as companion diagnostics, ancillary screening, prognostic, and treatment monitoring indicating global interest and significance of liquid biopsy in CRC (Table 1).

**Table 1:** Liquid biopsy assays currently in use for screening, diagnosis, and prognosis of Colorectal Cancer

Test Name (Ref)	Sample	Biomarker detected	Technology	Application
Epi proColon [9]	Plasma	SEPT9 methylation	Bisulfite converted DNA and PCR	Screening
OncoBEAM [10]	Plasma	34 RAS mutations- 16 mutations in KRAS codons 12, 13, 59, 61, 117, 146 and 18 mutations in NRAS codons 12, 13, 59, 61, 117, 146	Emulsion digital PCR with flow cytometry	Diagnostic
Idylla™ ctNRAS-BRAF-EGFR S492R Mutation Assay [11]	Plasma	18 mutations in NRAS exons 2,3,4 5 mutations in BRAF codon 600 2 mutations in EGFR codon 492	PCR	Diagnostic
AdnaTest ColonCancerSelect and AdnaTest ColonCancerDetect [12]	Blood	Colon-cancer-associated gene expression of GA733-2, CEA and EGFR	PCR	Diagnostic
Guardant360 [13]	Plasma	Tumour mutation profiling (73 genes)	NGS	Diagnostic
TruSight Oncology 500 portfolio [14]	Blood	pan-cancer comprehensive genomic profiling of Single nucleotide variants (SNVs), Indels, CNVs, fusions, and IO biomarkers (TMB, MSI)	NGS	Diagnostic
CellSearch [15]	Blood	CTC with CD45-, EpCAM + and (CK8, 18 and/or 19)	CTC immuno-isolation and detection by immune-fluorescence	Prognostic
Intplex [16]	Plasma	KRAS/NRAS/BRAF point mutations	PCR	Prognosis Treatment selection

*SEPT9* Septin 9,* GA733-2* Epithelial glycoprotein 40 gene, *CEA* Carcinoembryonic antigen,* EGFR* Epidermal growth factor receptor,* NGS* Next generation sequening, *TMB* Tumor mutational burden,* MSI* Microsatellite instability, *CNV* Copy Number Variations, *CTC* circulating tumor cells

**Fig. 1:**
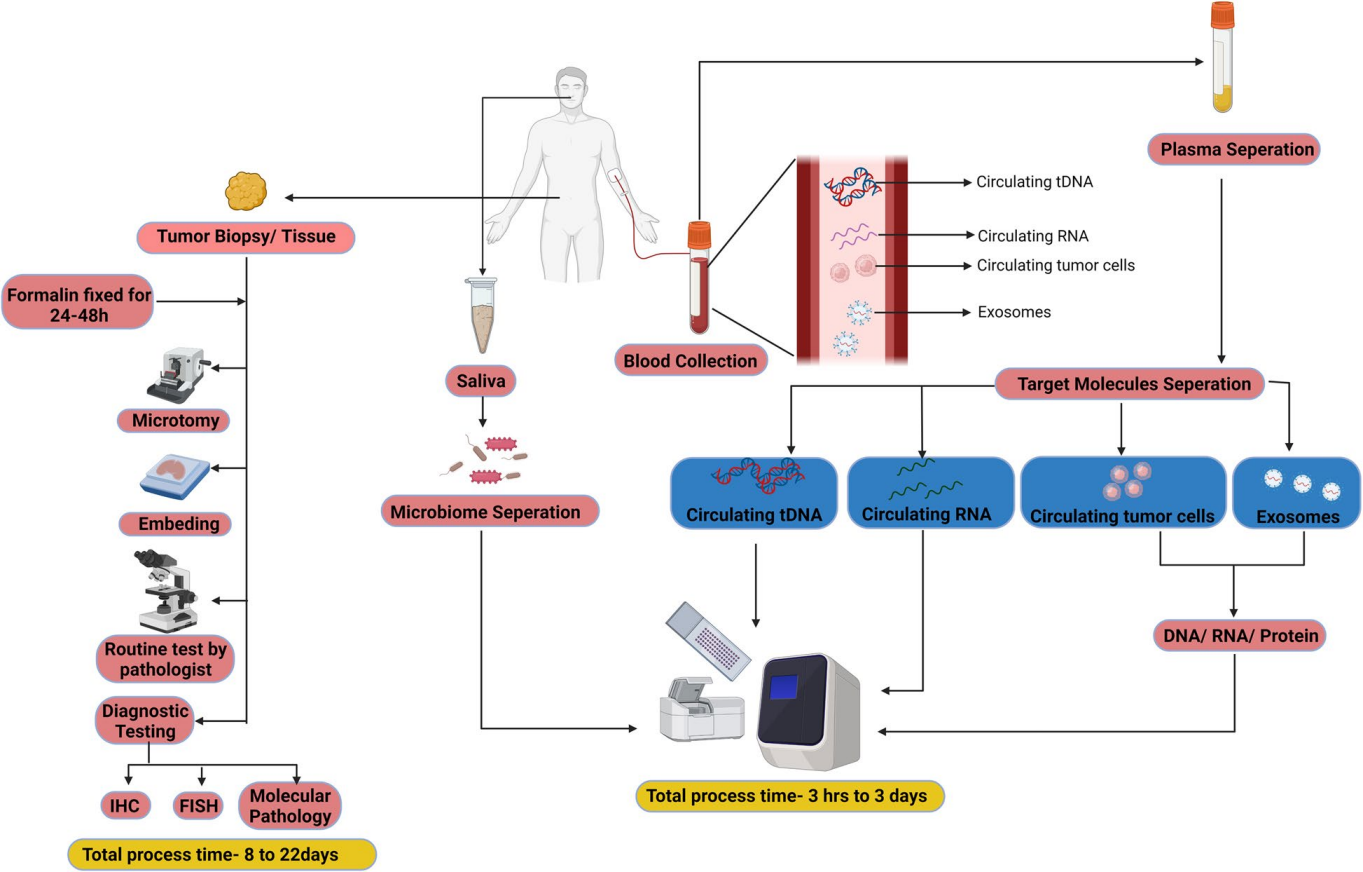
Comparison of tissue biopsy with liquid biopsy in colorectal cancer (CRC)

**Fig. 2:**
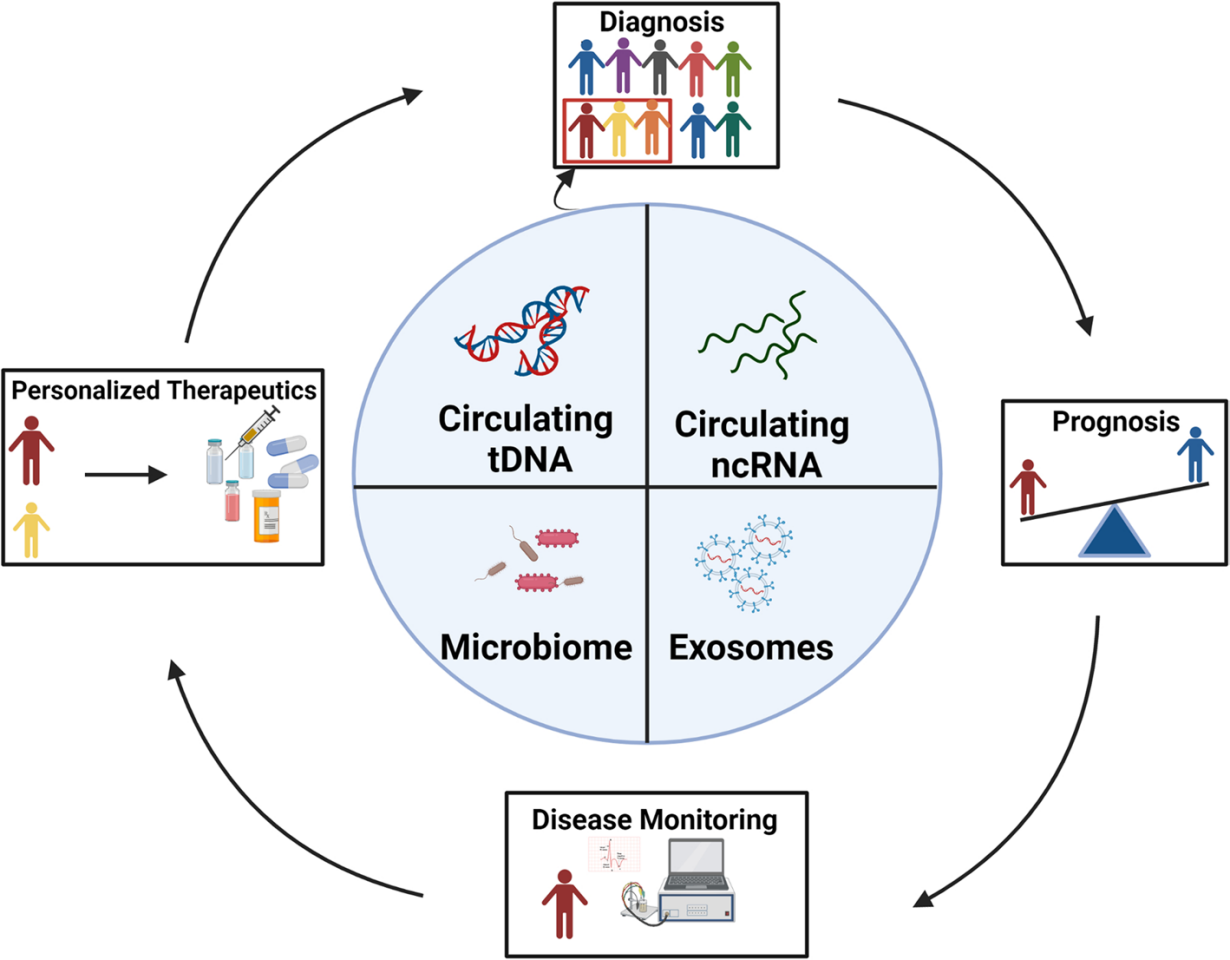
The utility of microbiome, exosomes, circulating ncRNAs and ctDNA in colorectal cancer

The major advantages of liquid biopsy in CRC is its ability to detect genetic/epigenetic landscape and tracking of genomic evolution/acquired resistance thus facilitating early diagnosis, disease progression and response to therapies [8, 17]. Furthermore, when compared to tissue biopsy, liquid biopsy confers several advantages including rapid turnaround time, ease of longitudinal/real-time monitoring of genetic heterogeneity, limited technical expertise and cost requirement for testing (Fig. 1) [17, 18]. With this perspective, liquid biopsy, could serve as an important modality in CRC management. Therefore, this review aims to discuss updated information on the liquid biopsy components including microbiome, exosomes, circulating noncoding (ncRNAs) and ctDNA for early detection, prognosis, and therapeutic monitoring in colorectal cancer (Fig. 2).

### Microbiome

The collection of microbes living in and on the human body is known as the microbiome and has been associated with cancer initiation, progression, and response to therapy [19]. In CRC, host-microbiota interactions are inevitable due to spatial proximity of the enteric milieu. Indeed, in CRC, the microbiota primarily causes a protumor microenvironment through their interaction with immune cells and subsequent release of inflammatory metabolites, or by inducing other specific metabolites and toxins. Detection of such markers, has been reported in the body fluids as a non-invasive, and cost-effective option for screening, diagnostic and prognostic strategies in CRC [20].

### Role of oral microbiota in CRC diagnosis

Metagenomic analysis of oral microbial dysbiosis to characterize diversity/richness of oral cavity-associated microbial species has been performed with reported among CRC, colorectal adenoma (CRA), and healthy controls (HC) [21]. Given the strong dependency of putative oral bacteria in gut colonization, Flemer et al., decoded the orally derived taxa involved in CRC development and proposed that such profiling could be used for CRC screening. The study identified eight differentially abundant oral microbiota operational taxonomic units (OTU) and formulated a random forest-based classification model, incorporating 16 oral microbiota OTUs to categorize CRC patients and healthy controls. The oral bacterial network analysis led to the conception of two influential co-abundance groups (CAGs) in CRC patients, namely oral pathogen CAGs with 7 OTUs and biofilm CAGs including 10 OTUs [22]. The oral pathogen CAGs such as *Fusobacterium, Porphyromonas, and Treponema* and the biofilm CAG such as *Streptococcus, Rothia, and Faecalibacterium* were documented to play a role in CRC pathogenesis while abundance of *Lachnospiraceae* CAG was negatively correlated with oral bacteria-led colonization, thus signaling a protective role in CRC pathogenesis. These results indicate that identification of CAGs could serve as useful markers for risk stratification in CRC [22]. Another study on 161 CRC, 34 CRA, and 58 controls reported dysbiosis of the oral pathogen CAGs: *Fusobacterium, Treponema and Porphyromonas* and the biofilm CAG; *Streptococcus, Faecalibacterium and Rothia* as important biomarkers for CRC pathogenesis. On the other hand, the study also validated five OTUs; *Porphyromonas, Streptococcus, Propionibacteriaceae, Cyanobacteria and Saccharibacteria* as oral microbiome biomarkers that can discriminate CRA and CRC patients from healthy controls [23]. Moreover, in several studies, CRC patients have exhibited a high relative abundance of OTUs including *Actinomyces odontolyticus, Haemophilus, Neisseria, Porphyromonas, Streptococcus,* and *Prevotella_7 genu, Peptococcus and Centipeda* in their salivary microbiota as compared to healthy controls. Some of these OTUs have been reported to be involved in CRC tumorigenesis [24, 25].

Comparative analyses on matched stool and saliva samples from CRC patients and healthy controls showed that four bacterial species: *Peptostreptococcus stomatis, Streptococcus anginosus, Solobacterium moorei, and Streptococcus koreensis* were significantly abundant in oral cavity of CRC patients [26]. Similarly, another study reported a decline in the relative abundance of the *Pasteurellaceae* family in controls while an increase of *Neisseriaceae* in CRC patients [27]. On the other hand, subgroup analyses showed that with increasing stages, the relative levels of *Proteobacteria* increased while those of *Bacillales* decreased, indicating the role of these salivary microbiomes in prognostication and staging of patients [27]. A nested case–control study on 231 CRC cases and 462 controls, suggested association of *Treponema denticola, Prevotella intermedia, Actinobacteria, Bifidobacteriaceae,Bacteroidetes, Prevotella denticola* and *Prevotella* sp. oral taxon 300 were associated with an increased CRC risk while *Prevotella melaninogenica, Firmicutes, Carnobacteriaceae, Streptococcaceae, Erysipelotrichaceae, Streptococcus, Solobacterium, Streptococcus* sp. oral taxon 058 and *Solobacterium moorei* were associated with a decreased risk of CRC indicating the utility of the oral microbiome markers with risk stratification [28]. Similarly, two separate studies documented a significant association of oral cavity derived *Lactobacillus*, *Rothia* and *Fusobacteria nucleatum* (*F.nucleatum*) strains with circulatory transmission and CRC colonization at tumor site [29, 30].

### Role of blood-based microbiota in CRC disease progression

Gut microbiome-associated metabolites in plasma/serum harbor promising potential in CRC detection, prognosis, and treatment monitoring. A study by Chen et al., reported a novel integrated approach of serum metabolomic analysis to discriminate CRC patients and adenomas from healthy controls. This panel known as gut microbiome-associated serum metabolites (GMSM) identified elevated levels of distinct microbiome-associated serum metabolites, including microbial metabolite N, O-Bis-(trimethylsilyl) phenylalanine in both serum and CRC tissues. Furthermore, change in these serum metabolites was positively associated with bacteria species, *C. bacterium* VE202-01 and *E. ramosum* indicating tumorigenesis-associated microbiome reprogramming in CRC patients. The sensitivity and specificity of GMSM was found to be 83.5% and 84.9% respectively indicating reliable diagnostic accuracy of these metabolites in CRC [31]. On the other hand, a retrospective study on 13,096 patients from Hong Kong showed the association between bacteremia and CRC incidence. Interestingly, the study reported that patients who had bacteremia with *Bacteroides fragilis, S.gallolyticus*, and *F.nucleatum* were majorly diagnosed with CRC as compared to the uninfected individuals. The authors postulated that these bacteria may have perturbed the intestinal barrier function, thus initiating dysbiosis, promotion of neoplastic lesions and eventually facilitating tumorigenic cycle in the colorectum [32]. In addition to this, anti-microbial antibodies can also serve as reliable markers for diagnosis. For e.g. a study by Wang et al., reported a significant difference in serum anti-*F. nucleatum* IgA (Immunoglobulin A) antibody levels in CRC patients as compared to HC/ benign cases thus suggesting the potential of *anti-F. nucleatum* antibodies as robust biomarker for CRC [33].

Detection of microbial translocation in blood of CRC patients has also shown prognostic significance. Dynamic alterations in genomic DNA encoding *16S rRNA, Escherichia coli, Bacteroides fragilis, Candida albicans*, was significantly correlated changes in circulating tumor cells (CTC) in metastatic CRC patients during three to six months of therapeutic window [34]. Similarly, to propose circulating bacterial DNA as biomarkers for CRC, Xiao et al., evaluated variation in bacterial species through whole-genome sequencing of plasma samples between CRC patients, colorectal adenoma (CRA) patients and healthy controls (HC). The authors recorded 28 distinct species with predictive potency for CRC compared to HC [35]. On the other hand, the association of genotoxic and enterotoxigenic strains of *Escherichia coli (E. coli)* and *Bacteroides fragilis (ETBF*) in CRC incidence was evaluated in a large study with 442 pairs of incident CRC cases and matched controls. The study documented that IgA and IgG dual-positivity to *E. coli* and ETBF were associated with an overall 1.79-fold higher odds of cancer progression [35]. Similarly, several studies have documented the seropositivity to *Helicobacter pylori (H.pylori) Vacuolating Cytotoxin (VacA)* and *S.gallolyticus (SGG)* pilus protein Gallo2178 as indicators of increased CRC risk [36, 37]. For e.g., a large study reported that anti-*H. pylori VacA* antibodies are associated with a significant 11% increased odds of CRC risk [38]. Similarly, sero-epidemiological data analyzing antibody responses to SGG pilus proteins evidence their role in CRC tumorigenesis [39–41]. Interestingly, Taylor et al. identified a type VII secretion system (T7SS) in SGG, designated as SggT7SS^T05^, that was identified as a contributor for colonization and promotion of CRC colon tumors [42]. Therefore, the evidence through literature evidences and establishes a crucial role of several bacterial species in CRC pathogenesis.

### Exosomes

Exosomes are nano-sized heterogeneous extracellular vesicles (EVs) enclosed by a lipoprotein bilipid layer that prevents its degradation and makes the cargo stable. These small vesicles enclose a variable spectrum of biologically active molecules, which mirror the composition of their originating cells, including nucleic acids (DNA, mRNA, miRNA, lncRNA, etc.), proteins and lipids [43].

Exosomes outperform other liquid biopsy components mainly because they exist in almost all body fluids (primarily blood, urine, cerebrospinal fluid, milk, saliva etc.) and possess high stability due to encapsulated cholesterol-rich lipid bilayer [44]. Additionally, since exosomes carry biological cargo from the living parental cells,they are more capable of representing their originating cells than ctDNA and CTCs [45]. Notably, during carcinogenesis, cancer cells often segregate and release various important deregulated proteins and other cellular components into the body fluids or blood stream (exosomes, proteins, and macromolecules) [46]. Several studies have found that cancer-derived exosomes are linked to tumor growth, development of metastatic niches, and immune evasion [47]. Therefore, isolation of exosomes from body fluids would be of major importance in early diagnosis, better prognosis and use of targeted therapeutic measures.

### Exosomes in CRC diagnosis

The differential expression of proteins such as those involved in deregulated fatty acid/amino acid metabolism pathways have been extensively reported in body fluids derived exosomes of CRC patients [48]. A study by Chen et al., demonstrated clinical importance of exosomal cargo by reporting upregulation of 36 proteins, involved in modulation of metastatic environment, and downregulation of 22 proteins involved in tumor cell proliferation/survival in exosomes of CRC patients [49]. Similarly, a recent study identified exosomal cargo protein QSOX1 to be significantly reduced in plasma derived exosomes from CRC patients as compared to controls with demonstrated Area Under Curve (AUC) of 0.904, indicating that deregulated exosomal proteins can serve as promising novel biomarker for early diagnosis and non-invasive risk stratification in CRC [50]. On the other hand, exosomes are enriched in cell surface proteins and their upregulated expression has also been explored as diagnostic biomarkers in CRC. For e.g. a study observed that Glypican-1(GPC1) is tenfold higher in plasma derived exosomes of CRC patients as compared to healthy controls [51]. Similarly, CopineIII (CPNE3) protein was found to be upregulated in the plasma derived exosomes of CRC patients and significant correlation was observed between CPNE3 expression in CRC tissues and matched serum exosomal CPNE3 expression indicating its utility in non-invasive CRC screening/diagnosis [52].

Several exosomal miRNAs have also been found differentially expressed in CRC patients and reported as potential biomarkers for the CRC clinical diagnosis [53–55]. A study by Karimi et al. identified 11 miRNAs and found that out of these 11 miRNAs, the expression of miR-23a and miR-301a was significantly higher in serum exosomes from CRC patients as compared to healthy controls [56]. In another study, miR-17-92a and miR-19a were found to be upregulated in CRC patients and their overexpression was correlated with early tumorigenesis, cancer proliferation and CRC tumor invasion [57, 58]. Additionally, few long non-coding RNAs (lncRNAs), have also been reported to be expressed differently in exosomes of CRC patients. For e.g., downregulation of lncRNA urothelial cancer associated 1 (UCA1) while upregulation of Circular RNA (circRNA) homeodomain interacting protein kinase 3 (HIPK3) was reported in serum derived exosomes in CRC patients [59]. Similarly, Colorectal neoplasia differentially expressed-h (CRNDE-h) lncRNA, was found to be upregulated in 148 CRC patients when compared with controls. Moreover, a relative expression cut-off value of 0.020 for exosomal CRNDE-h was determined for distinguishing CRC diseased group from controls (sensitivity of 70.3% and specificity of 94.4%) indicating the promise of exosomal lncRNAs in the clinical diagnosis of CRC [60].

### Exosomes in CRC Prognosis

Several studies have reported the role the exosomes in regulating CRC progression. Accumulating data indicates potential role of exosomal cargo in poor prognosis of CRC through mediating cross talk between CRC cells, fibroblast, and macrophage phenotypes. In lieu of this, several studies highlights the diagnostic and prognostic importance of exosomes in CRC [61]. For instance, exosomal dipeptidyl peptidase IV (DPP4) has been identified as an inducer of angiogenesis via activation of SMAD signaling pathway and its inhibition has been shown to suppresses tumor growth in vivo. Therefore, elevated levels of DPP4 can be related to progression and metastasis. A study on 5-Fluorouracil (5-FU) resistant colon cancer observed that an enzymatically active form of DPP4 is secreted into the microenvironment by various body fluids and high expression of DPP4 in poorly differentiated colon cancer tissues indicates that DPP4 activity in bodily fluids might be an efficient non-invasive diagnostic biomarker in CRC [62].

Recently, it has been reported that over expression of serum exosomal microRNAs (miRNAs) correlate positively with tumor progression and liver metastasis in CRC. For e.g. higher expression of miR-193a and miR-25-3p, miR-17-5p and miR-92a-3p, miR-21, miR-203 are reported to promote metastasis to liver by inducing vascular permeability/angiogenesis and have been suggested as independent predictors of poor prognosis in CRC patients [63–67]. However, more research into the probable processes underpinning their participation in metastasis is needed.

### Exosomes in CRC therapeutics

Oxaliplatin (Oxa) and 5-fluorouracil (5-Fu) are the commonly used chemotherapeutic drugs for CRC treatment but chemoresistance of these drugs is the major obstacle for effective outcome [68]. Thus, an urgent need of biomarkers is required to differentiate patients with drug sensitivity from those with drug resistant. Several studies have assessed the correlation between exosomal cargo and chemoresistance in CRC patients and hence, exosomes are considered to play an important role in treatment response assessment [69–71]. For instance, CRC associated lncRNA (CCAL) in the exosomes secreted by CRC-associated fibroblasts (CAFs) were reported to promote Oxa resistance of CRC cells by activating β-catenin pathway [72]. Additionally, exosomal Wnts derived from cancer associated fibroblasts (CAFs) and upregulation of miR-196b-5p in CRC serum exosomes were found to promote chemoresistance to 5-FU [73, 74]. Recently, a panel of miRNAs including miR-96-5p, miR-1229-5p, miR-21-5p and miR-1246 in serum exosomes of CRC patients were observed to be upregulated in 5-FU CRC resistant patients than chemo-sensitive controls [75]. In addition to treatment monitoring, exosomes can also serve as vehicles to deliver drugs to the target cells in various cancers including CRC. For example, in one of the recent studies, miR-21i (miR-21 inhibitor oligonucleotide) and 5-FU were simultaneously delivered to CRC resistant cells using engineered exosomes that effectively reversed drug resistance and enhanced the treatment efficacy [71]. Additionally, it has been reported that poor response to Oxaloplatin was seen in CRC patients with low expression of miR-128-3p. The treatment response was improved by transporting miR-128-3p containing exosomes derived from transfected normal intestinal FHC cells to Oxa-resistant cells both in vitro and in vivo [76]. In one of the studies, A33 antibody -functionalized exosomes loaded with doxorubicin (Dox) were used to target CRC cells that resulted in tumor growth inhibition [77]. Thus, it is evident that exosomes represent promising sources of non-invasive biomarkers for diagnosis, prognosis, and treatment of CRC.

### Circulating noncoding RNAs in CRC

#### Circulating small non-coding piRNAs for CRC diagnosis and prognosis

In recent years, circulating small non-coding RNAs, known as PIWI-interacting RNAs (piRNAs) have gained attention as novel non-invasive biomarkers for disease diagnosis and prognosis. piRNAs are 24–32 nucleotides in length classified into transposon-derived piRNAs, mRNA-derived piRNAs and lncRNAs-derived piRNAs [78]. They are known to specifically interact with the PIWI protein subfamily of the ARGONAUTE family and have been associated with several gene regulation mechanisms including transposon silencing, epigenetic programming, DNA rearrangements, mRNA turnover, and translational control [79].

In CRC, dysregulated levels of piRNAs have been associated with DNA methylation and T cell regulation. To date, only few circulating piRNAs have been identified in CRC. However, their altered expressions have been suggested as potential candidates as diagnostic/prognostic biomarkers. For example, serum piR-823 levels have been positively correlated with CRC staging with significantly higher levels observed with advanced stages (III and IV) [80]. Similarly, another study observed that downregulated expression of piRNA-28876 and piRNA-5937 correlated with in early-stage (TNM stages I-II) indicating the role of these piRNAs in CRC staging [81]. Interestingly, a study by Vychytilova-Faltejskova et al. identified piRNAs; piR5937, piR-28876, piR-23210, piR-32159 to be significantly downregulated in serum samples with piR5937 and piR-28876 found specifically only in CRC patients indicating further investigation of these markers as potential candidates for CRC diagnosis [82]. Moreover, a study by Yin et al. evidenced that the suppression of piR-823 expression suppressed cell proliferation, induced apoptosis, and cell cycle arrest in G1 phase in CRC cell lines HCT116 and DLD-1 evidencing the significance of piR-823 in CRC. Furthermore, the authors also observed that the expression of piR-823 was significantly upregulated in CRC tissues as compared to adjacent tissues [83]. In addition to this, two separate studies, investigating piR-1245 and piR-24000 have documented that the overexpression of these piRNAs correlates with poor differentiation, presence of distant metastases and higher stage [84]. Additionally, it was observed that piR-1245 repressed expression tumor suppressor genes involved in cell proliferation and apoptosis indicating the role of these piRNAs in disease modulation [85].

Using high-throughput sequencing (HTS), Qu et al., identified five differentially expressed serum piRNAs; piR-001311, piR-004153, piR-017723, piR-017724, piR-020365, in CRC patients as compared to healthy controls. The study specifically observed that downregulation of piR-017724 was associated with overall/progression-free survival in CRC [86]. A study by Mai et al. observed significantly upregulated expression of piR-54265 in serum samples from CRC patients. Mechanistically, piR-54265 binds specifically to Piwi Like RNA-Mediated Gene Silencing 2 (PIWIL2) protein, thus, forming PIWIL2/STAT3/phosphorylated-SRC complex which facilitates proliferation and metastasis by STATS3 phosphorylation. Therefore, piR-54265 could serve as an important biomarker of diseases modulation and dynamics [87, 88]. Taken together, these findings indicate that these piRNAs could serve as diagnostic and prognostic biomarkers as well as potential therapeutic targets for colorectal cancer.

In addition to non-coding piRNAs, several other noncoding RNAs including miRNA, Long noncoding RNAs and circular RNAs have been extensively studied and associated with various biological functions ranging from being as microRNAs sponges, RNA-binding proteins, regulating transcription, and encoding for peptides (Ref). In view of CRC, these ncRNAs are studied for their utility as prognostic, diagnostic, and therapeutic biomarker. Due to lack of space, the role of some of these biomarkers has been discussed in Tables 2 and 3 respectively.

**Table 2:** Expression of circulating LncRNA and circRNA in colorectal cancer (CRC)

LncRNAs				
	**Expression in CRC**	**Clinical relevance**	**Sample type**	**Ref**
DANCR	upregulated	Prognosis	Serum	[89]
LncRNA-ATB	upregulated	Diagnosis / therapy	serum	[90]
CCAT1	upregulated	Diagnosis / therapy	Serum	[90]
B3GALT5-AS1	downregulated	Diagnosis	Serum	[91]
MEG3	downregulated	Prognosis	Serum	[92]
SNHG11	Upregulated	Diagnosis/therapy	Plasma	[93]
LncRNA RP11909B2.1	downregulated	Diagnosis	Serum	[94]
***circRNAs***				
hsa_circ_0001900, hsa_circ_0001178, hsa_circ_0005927	Upregulated	Diagnosis	Plasma	[95]
circ-CCDC66, circ-ABCC1, circ-STIL	Downregulated	Diagnosis	Plasma	[96]
circ-VAPA	Upregulated	Diagnosis/therapy	Plasma	[97]
hsa_circ_0082182, hsa_circ_0000370	Upregulated	Diagnosis	Plasma	[98]
hsa_circ_0035445	Downregulated	Diagnosis	Plasma	[98]
hsa_circ_0004831	Upregulated	Prognosis	Serum	[99]
hsa_circ_0007534	Upregulated	Prognosis	Plasma	[100]
hsa_circ_0001649	Downregulated	Diagnosis	Serum	[101]
hsa_circ_0002320	Downregulated	Diagnosis/Prognosis	Plasma	[102]
circ_PVT1, circ_001569	Upregulated	Diagnostic/ Prognosis	Plasma	[103]
hsa_circ_0004585	Upregulated	Diagnostic	Plasma	[104]

*DANCR* Differentiation antagonizing non-protein coding RNA, *LncRNA-ATB* Long Noncoding RNA Activated By TGF-Beta, *CCAT1* Colon Cancer Associated Transcript 1, *B3GALT5-AS1* β 1, 3 galactosyltransferase 5 Antisense RNA 1, *MEG3* Maternally Expressed Gene 3, *SNHG11* Small Nucleolar RNA Host Gene 11, *lncRNA RP11-909B2.1* Long non-coding intergenic RNA

**Table 3:** Expression of circulating miRNAs in CRC

miRNAs	Expression in CRC	Biomarker	Sample type	Ref
miR-21	Upregulated	Diagnostic	Plasma, Saliva	[105]
miR-30e-3p, let-7d-5p, let-7a-5p, let-7f-5p miR-375, miR-486-3p, miR-486-5p, miR-1180-3p	Upregulated Downregulated	Diagnostic	Serum	[106]
miR-92a-3p	Upregulated	Diagnostic	Plasma, Serum	[107]
miR-762	Upregulated	Diagnostic	Serum	[53]
miR-211, miR-25	Upregulated	Diagnostic	Plasma	[54]
miR-618	Upregulated	Prognostic	Serum	[108]
miR-21, miR-145, miR-203, miR-155, miR-210, miR-31, miR-345	Upregulated	Diagnostic	Plasma	[109]
miR-28-3p, let-7e-5p, miR-106a-5p, miR-542-5p	Upregulated	Diagnostic	Plasma	[55]
miR-449a	Downregulated	Diagnostic, Prognostic	Plasma	[110]
miR-29c, miR-149	Downregulated	Diagnostic	Serum	[111]
miRNA-223	Upregulated	Diagnostic	Serum	[112]
miR-19a-3p, miR-203-3p, miR-221-3p, and let-7f-5p	Upregulated	Diagnostic	Serum	[113]
miR-30e-3p, miR-146a-5p miR-148a-3p	Upregulated Downregulated	Diagnostic	Serum	[114]
miR-451a	Upregulated	Diagnostic	Serum	[115]
miR-21, miR-23a, miR-27a	Upregulated	Diagnostic	Serum	[116]
miR-584-5p, miR-15b-5p, miR-425-3p	Upregulated	Diagnostic	Plasma	[117]
miR-944	Downregulated	Diagnostic	Serum	[118]
miR-21, miR-92a	Upregulated	Diagnostic	Plasma	[119]
miR-592	Upregulated	Diagnostic	Serum	[120]

### Circulating tumor DNA (ctDNA) and circulating tumor cells (CTC)

Circulating cell free DNA (ccfDNA) are single and double stranded DNA fragments (≤ 200pb) released into the body fluids by apopotic/necrotic cells. Their presence is indicative of various pathological conditions including inflammation, autoimmune diseases, or malignancies [121]. In context of malignancies, the ccfDNA released by tumor cells is known as circulating tumor DNA (ctDNA) and accounts for 0.01–90% of the total ccfDNA in the blood [121].

In CRC, ctDNA is considered an important liquid biopsy component as it carries several genetic variations (copy number variations, single nucleotide polymorphisms and mutations) that have similar genetic make-up as their intra-tumoral parent tissue [121]. Indeed, the concordance of cancer specific genetic and epi-genetic alterations observed in ctDNA and matched CRC tumor tissue has been demonstrated in several studies indicating the utility of ctDNA in determining complementary genetic information on primary and metastatic tumors without the need for invasive tissue biopsy [122–125].

### ctDNA for CRC screening and diagnosis

The change in the levels of ccfDNA in body fluids has been suggested as an important screening tool in CRC. Clear-cut difference in ccfDNA levels between CRC diseased and healthy controls have been documented in several studies [126–129]. For e.g. a study conducted on 158 plasma samples from disease positive and negative groups showed that low level (< 10 ng/ml) of ccfDNA were observed in disease negative group while elevated ccfDNA concentration (1.180 to 321 ng/ml) was detected in CRC patients [130]. Furthermore, the study also determined an optimal ccfDNA concentration cutoff of 7.0 ng/ml as sensitive and specific concentration to discriminate diseased from control groups [130]. Though an interesting finding, there are several limitations to this approach. For e.g. benign lesions (having similar mutations as cancer cells) are also known to release ccfDNA into the blood stream and may therefore contribute to a non-specific increase in ccfDNA levels thus giving false positive results [131]. In addition to this, factors such as tumor burden, metastatic sites and cellular turnover can also influence ccfDNA levels in body fluids. In lieu of these limitations, several studies have tested ctDNA fragment length, instead of ccfDNA levels, as a screening tool in CRC. A comparative genome wide study on 27 patients showed that the DNA fragmentation profile of CRC patients was significantly altered as compared to healthy controls [132]. However, the authors further suggested that since ctDNA fragment length profiling may be influenced by clinical characteristics and methylation changes, therefore additional diagnostic approaches should be tested in combination with ctDNA fragment length to fully elucidate its role as a screening tool in CRC.

DNA methylation is one of the most important and earliest epigenetic hallmarks of CRC [133]. Up to now, clinical studies have shown that the methylated epigenetic ctDNA markers serve as promising candidates for CRC screening modality in serum/plasma of CRC patients [134]. A study by Young et al., designed a multi-panel methylation assay for detection of specific methylation markers, Branched Chain Amino Acid Transaminase 1(BCAT1), IKAROS Family Zinc Finger 1I(KZF1) and Interferon Regulatory Factor 4 (IRF4), in plasma of CRC patients. The study reported that this panel was able to differentiate CRC patients from controls with a specificity of 94.1% and sensitivity of 71.2%. In addition to this, the detection rate of targeted methylated genes was also found to increase significantly with increasing stage indicating the significance of these cfDNA methylation markers as screening and diagnostic tools in CRC [135]. On the other hand, several markers such as MutL homolog 1(MLH1) promoter methylation markers [136] and Septin 9 (SEPT 9) [137] have also been identified as suitable markers with high sensitivities (78% and 87%) and specificities (100% to 90.6%) to differentiate CRC patients from heathy controls. In addition to this, two separate studies have documented that Secretin Receptor (SCTR) hypermethylation and Transmembrane Protein 240 (TMEM240) promoter hypermethylation is detected only in cfDNA samples of CRC patients but not in healthy controls [138]. For e.g. the study observed that the median DNA methylation levels of TMEM240 promoter hypermethylation is 0.0021 in CRC patients while it is 0.0000 in healthy controls [139]. These results indicate that several methylation markers circulating in cfDNA have a potential role in CRC screening and as such these should be explored further.

CRC staging is an important diagnostic parameter and plays a significant role in treatment management. It is usually done on tissue biopsy samples and is therefore associated with several limitations. However, several studies on cfDNA methylation markers have evidenced on the role of cfDNA in CRC staging. A study using methylated CpG tandem amplification and sequencing (MCTA-Seq) method, discovered a panel of 80 methylation markers that could discriminate CRC stage I, II, III/IV patients from healthy controls with a sensitivity of 62%, 81%, 85%, and 79% respectively and a specificity of 86% [140]. Moreover, a cfDNA methylation model, based on 11 DNA methylation biomarkers, was established and tested for the differentiation of early-stage and advanced CRC adenomas with a sensitivity and specificity of 87.9% and 76.5% respectively [141]. Similarly, a study by Jensen et al., defined three tumor-specific DNA methylation markers, known as Chromosome 9 Open Reading Frame 50 (C9orf50), Potassium Voltage-Gated Channel Subfamily Q Member 5 (KCNQ5) and CAP-Gly Domain Containing Linker Protein Family Member 4 (CLIP4) in circulating cell-free DNA. These markers were shown to effectively discriminate CRC tumor stages with a specificity of 99% and sensitivities of 80% in stage I, 85% in stage II, 89% in stage III and 88% in stage IV [142]. The results from these studies indicate the utility of cfDNA methylation markers as valuable non-invasive tools for CRC screening and diagnosis.

### ctDNA as predictor of response and treatment resistance/recurrence

In addition to screening and diagnosis, ctDNA has also been reported as a sensitive predictor of response, emergence of resistance and disease recurrence in CRC. Indeed, detection of ctDNA post-surgery has been associated with prognostic utility. For e.g. a study reported that patients with detectable ctDNA post-surgery have a greater 5-year recurrence risk (38.6% vs 85.5%) and poorer overall survival (64.6% vs 89.4%) as compared to patients with undetectable ctDNA [143]. Similarly, in chemotherapy treated patients, a reduction in ctDNA levels, up to ≤ 50%, at 2–8 weeks after initiation of chemotherapy, is a predictor of treatment response with significant correlation observed between changes in ctDNA level and progression-free/overall survival in metastatic CRC patients [144]. Moreover, detection of Rat sarcoma virus (RAS), Epidermal Growth Factor Receptor (EGFR), and B-Raf and v-Raf murine sarcoma viral oncogene homolog B (BRAF) mutations in ctDNA can serve as predictors of response in EGFR treated metastatic CRC patients. Accordingly, patients with undetectable ctDNA mutations in RAS/BRAF were reported to show a better response to anti-EGFR treatment than those with detectable mutations at baseline [145, 146]. On the other hand, a concordance of 93% was observed between the detected ctDNA mutations with respective tumor tissue indicating the utility of ctDNA for longitudinal genomic profiling during treatment to evaluate the development of resistance [147].

In addition to EGFR treatment, immune checkpoint blockade (ICB) with anti-Programmed cell death protein 1(anti-PD-1)/anti- Programmed death-ligand 1 (anti-PD-L1) has shown beneficial results in microsatellite instable (MSI) high patients. In some cases, immunotherapy is also given to microsatellite stable patients (MSS) when all treatment lines fail. [148]. Given the expense associated with immunotherapy, particularly with low benefit for MSS patients, identification of early biomarker of response is important to cut costs and decrease potential toxicities [148]. A study by Wang et al., observed that in MSS CRC patients, a decline in ctDNA was associated with initial response and radiographic disease stabilization while a rise of ctDNA at 4 weeks post treatment predicted tumor progression at 2 months [148]. Similarly, a recent study on 23 deficient MisMatch Repair (dMMR)/microsatellite instability-high (MSI-High) patients observed that serial circulating tumor DNA (ctDNA) monitoring during Programmed cell death protein 1 (PD-1) blockade/progression was able to predict responses weeks ahead of standard imaging indicating that changes in ctDNA can help predict early tumor response to immunotherapy [149].

### Circulating tumor cells

Circulating tumor cells (CTCs) are cells shed from a primary tumor into the vasculature with an intact viable nucleus, a positive cytokeratin, epithelial cell adhesion molecule (EpCAM) and a negative CD45 molecule [150]. CTCs are detected in metastatic carcinomas but are extremely rare in healthy controls/nonmalignant disease [151]. In lieu of this, they are considered as seeds for the growth of metastatic tumors in distant organs of the body and therefore their detection as liquid biopsy markers are vital from prognostic and therapeutic monitoring perspective. However, limitations such as low frequency ~ 1–10 CTCs/mL of whole blood, difficulty in isolation of single CTC from the background of all other blood components, short half-life of CTCs in the bloodstream (1–2.4 h), downregulation of CTC associated markers and heterogeneity are major technical challenges for this liquid biopsy component [150]. Albeit all these limitations, several studies have shown that the phenotypic and molecular characteristics of CTCs are valuable for identifying specific genetic mutations for CRC diagnosis, prognosis and treatment dynamics [152–154].

### Circulating tumor cells in CRC diagnosis/prognosis

Several studies have evidenced that differences in CTC counts can be used in CRC for distinguishing patients with benign disease and those with colorectal cancer. A study by Yang et al. reported that CTC counts were significantly higher in colorectal cancer patients as compared to patients with colorectal polyps. On the other hand, CTC counts also varied according to anatomical locations and tissue differentiation with poorly differentiated tumors showing higher counts. Location wise, highest counts were observed in sigmoid tumors, followed by rectal tumors, ascending, transverse and descending colon indicating the role of CTC counts in diagnosis/screening [155]. Similarly, a study on stage I–IV CRC, adenomas and healthy controls showed that mean CTC counts were highest in stage IV CRC patients and low in adenomas and healthy controls. The study evidenced that CTC counts could differentiate between healthy and adenoma groups as well as patients in various stages of cancer indicating the diagnostic value of CTC counts in CRC [156].

The role of CTC counts in CRC prognosis was investigated in a prospective study on 149 CRC patients (79 colon cancer, 70 rectal cancer) undergoing surgical intervention. It was reported that high CTC counts correlated significantly with increased tumor stage with differences observed in T stage and N stages of the left and right hemicolon cancer. Furthermore, high CTC counts correlated with high tumor recurrence rate and worse prognosis indicating that change in CTCs counts could serve as a valuable tool for CRC prognosis [157]. Similarly, dynamic monitoring of CTC counts as a prognostic tool was investigated in a prospective phase II trial on153 CRC patients with resectable liver metastasis (LM). The enrolled patients were treated with combination of first-line triplet/doublet chemotherapy combined with targeted therapy. CTC counts were performed at various times lines including baseline, after 4 weeks of therapy and before surgery. It was reported that elevated CTC counts in 19% of patients decreased during therapy and before surgery indicating the prognostic value of CTC in CRC [158].

### Circulating tumor cells in CRC treatment

CTC counts and its associated biomarkers have been evaluated and documented as valuable tools for monitoring treatment dynamics in CRC. A large-scale prospective trial (CORIOLAN Trial) 47 on unresectable advanced CRCs patient’s refractory to standard treatments reported the prognostic value of CTCs in CRC prognosis. In this study, CTC counts were taken at day 1 and day 15 of treatment and the results showed that patients with high CTC counts at baseline had worse overall survival [159]. Similarly, a prospective randomized phase III trial (VISNÚ-1 trial), evaluated CTC counts as predictor of response in 349 metastatic colorectal cancer patients treated with 5-Fluorouracil/leucovorin, oxaliplatin, irinotecan (FOLFOXIRI) plus bevacizumab. The results showed that patients with CTC ≥ 3 on FOLFOXIRI plus bevacizumab had a significantly higher median progression free survival as compared to 5-fluorouracil/leucovorin and oxaliplatin plus bevacizumab group indicating the role of CTCs in treatment selection [160].

On the other hand, a study on 30 patients with locally advanced rectal cancer reported that monitoring of protein markers such as thymidylate synthase (TYMS) and excision repair protein, RAD23 homolog B (RAD23B) in CTCs can help to predict resistance to chemotherapy/radiotherapy. The study reported the expression of TYMS and RAD23B in 83% and 75% of non-responders. Furthermore, TYMS was found to be absent in patients with pathologic complete response indicating that TYMS/RAD23B expression in CTCs can help to distinguish responding and non-responding patients in CRC [161]. Similarly, a study by Shou et al. in 50 relapsed patients with stage III or stage IV CRC patients identified a novel six-gene assay (CEA, EpCAM, Cytokeratin 19 (CK19), Mucin 1(MUC1), EGFR and tyrosine-protein kinase Met (C-Met markers) in CTC indicating the role of these markers as effective predictors of progression-free survival in CRC [162].

## Conclusion

Liquid biopsy has emerged as a clinically useful tool approval of several test solutions indicating the utility of liquid biopsy as futuristic approach for timely and personalized therapeutic decisions. In this review, we have provided updated information on various novel liquid biopsy markers such as a) oral microbiota related bacterial network b) gut microbiome-associated serum metabolites c) PIWI-interacting RNAs (piRNAs), microRNA(miRNAs), Long non-coding RNAs (lncRNAs), circular RNAs (circRNAs) and d) circulating tumor DNAs/circulating tumor cells for their role in disease diagnosis, prognosis, and treatment monitoring in CRC. However, larger clinical studies/clinical trials on these markers are needed in to understand and validate the translation of these liquid biopsy components into high throughput applicable solutions in clinical settings. Gathering evidence on these aspects will power the applicability of liquid biopsy for accurate, personalized, and specific guidance on CRC management.

## Data Availability

Not applicable.

## Abbreviations

piRNAs: PIWI-interacting RNAs
miRNAs: MicroRNA
lncRNAs: Long non-coding RNAs
circRNAs: Circular RNAs
ctDNA: Circulating Tumor DNAs
CTC: Circulating Tumor Cells
CT: Computed Tomographic
FIT: Fecal Immunochemical Test
gFobt: Guaiac-based fecal occult blood test
NDRG4: N-myc downregulated gene 4
BMP3: Bone Morphogenetic Protein 3
KRAS: Kirsten Rat Sarcoma Virus
AA: Advanced Adenomas
mSEPT9: Methylated Septin 9
CEA: Carcinoembryonic antigen
CA19-9: Carbohydrate antigen 19–9
ncRNAs: Circulating noncoding RNA
CRA: Colorectal Adenoma
HC: Healthy Controls
OUT: Operational Taxonomic Units
CAGs: Co-abundance Groups
GMSM: Gut Microbiome-associated Serum Metabolites
T7SS: Type VII secretion system
GPC1: Glypican-1
CPNE3: CopineIII
DPP4: Dipeptidyl Peptidase IV
5-FU: 5-Fluorouracil
Oxa: Oxaliplatin
CAFs: Cancer Associated Fibroblasts
BCAT1: Branched Chain Amino Acid Transaminase 1
KZF1: IKAROS Family Zinc Finger 1I
IRF4: Interferon Regulatory Factor 4
MLH1: MutL homolog 1
SEPT 9: Septin 9
SCTR: Secretin Receptor
TMEM240: Transmembrane Protein 240
C9orf50: Chromosome 9 Open Reading Frame 50
KCNQ5: Potassium Voltage-Gated Channel Subfamily Q Member 5
CLIP4: CAP-Gly Domain Containing Linker Protein Family Member 4
RAS: Rat Sarcoma Virus
EGFR: Epidermal Growth Factor Receptor
BRAF: B-Raf and v-Raf murine sarcoma viral oncogene homolog B
ICB: Immune Checkpoint Blockade
anti-PD-1: Anti-Programmed cell death protein 1
anti-PD-L1: Anti- Programmed death-ligand 1
dMMR: Deficient MisMatch Repair
MSI-High: Microsatellite Instability-high
EpCAM: Epithelial Cell Adhesion Molecule
LM: Liver metastasis
TYMS: Thymidylate Synthase
RAD23B: RAD23 homolog B
CK19: Cytokeratin 19
MUC1: Mucin 1
C-Met: Tyrosine-protein kinase Met

## References

[1] Sung H, Ferlay J, Siegel RL, Laversanne M, Soerjomataram I, Jemal A (2021). Global Cancer Statistics 2020: GLOBOCAN Estimates of Incidence and Mortality Worldwide for 36 Cancers in 185 Countries. CA Cancer J Clin.

[2] Organization WH (2020). lobal Health Estimates 2020: Deaths by Cause, Age, Sex, by Country and by Region, 2000–2019.

[3] Liu Y, Zhao G, Miao J, Li H, Ma Y, Liu X (2020). Performance Comparison Between Plasma and Stool Methylated SEPT9 Tests for Detecting Colorectal Cancer. Front Genet.

[4] Thrumurthy SG, Thrumurthy SS, Gilbert CE, Ross P, Haji A (2016). Colorectal adenocarcinoma: risks, prevention and diagnosis. BMJ..

[5] Society AC. Colorectal Cancer Facts & Figures 2020–2022 2020 [Available from: https://www.cancer.org/content/dam/cancer-org/research/cancer-facts-and-statistics/colorectal-cancer-facts-and-figures/colorectal-cancer-facts-and-figures-2020-2022.pdf.

[6] Hamzehzadeh L, Yousefi M, Ghaffari SH (2017). Colorectal Cancer Screening: A Comprehensive Review to Recent Non-Invasive Methods. Int J Hematol Oncol Stem Cell Res.

[7] Baxter N, Rabeneck L (2009). New findings about the risks and limitations of colonoscopy used in the early detection of colorectal cancer. Healthc Q.

[8] Fernandez-Lazaro  D, Garcia Hernandez JL, Garcia AC, Cordova Martinez A, Mielgo-Ayuso J, Cruz-Hernandez JJ (2020). Liquid Biopsy as Novel Tool in Precision Medicine: Origins, Properties, Identification and Clinical Perspective of Cancer's Biomarkers. Diagnostics (Basel).

[9] Epi proColon® [Available from: https://www.epiprocolon.com/us/.

[10] OncoBEAM™ [Available from: https://sysmex-inostics.com/products/onco-beam/.

[11] IdyllaTM ctNRAS-BRAF-EGFR S492R Mutation Assay.

[12] AdnaTest ColonCancer.

[13] Guardant360® CDx [Available from: https://guardant360cdx.com/.

[14] TruSight Oncology 500 High-Throughput [Available from: https://www.illumina.com/products/by-type/clinical-research-products/trusight-oncology-500-ht.html.

[15] CELLSEARCH® [Available from: https://www.cellsearchctc.com/.

[16] IntPlex® [Available from: https://diadx.com/.

[17] Yamada T, Matsuda A, Koizumi M, Shinji S, Takahashi G, Iwai T (2019). Liquid Biopsy for the Management of Patients with Colorectal Cancer. Digestion.

[18] Pantel K, Alix-Panabieres C (2019). Liquid biopsy and minimal residual disease - latest advances and implications for cure. Nat Rev Clin Oncol.

[19] Sepich-Poore GD, Zitvogel L, Straussman R, Hasty J, Wargo JA (2021). Knight R. The microbiome and human cancer. Science.

[20] Parisi A, Porzio G, Cannita K, Ficorella C, Mattei V (2021). What Is Known about Theragnostic Strategies in Colorectal Cancer. Biomedicines.

[21] Thomas AM, Manghi P, Asnicar F, Pasolli E, Armanini F, Zolfo M (2019). Metagenomic analysis of colorectal cancer datasets identifies cross-cohort microbial diagnostic signatures and a link with choline degradation. Nat Med.

[22] Flemer B, Warren RD, Barrett MP, Cisek K, Das A, Jeffery IB (2018). The oral microbiota in colorectal cancer is distinctive and predictive. Gut.

[23] Zhang S, Kong C, Yang Y, Cai S, Li X, Cai G (2020). Human oral microbiome dysbiosis as a novel non-invasive biomarker in detection of colorectal cancer. Theranostics.

[24] Kageyama S, Takeshita T, Takeuchi K, Asakawa M, Matsumi R, Furuta M (2019). Characteristics of the Salivary Microbiota in Patients With Various Digestive Tract Cancers. Front Microbiol.

[25] Wang Y, Zhang Y, Qian Y, Xie YH, Jiang SS, Kang ZR, et al. Alterations in the oral and gut microbiome of colorectal cancer patients and association with host clinical factors. Int J Cancer. 2021. 10.1002/ijc.33596 33844851

[26] Uchino Y, Goto Y, Konishi Y, Tanabe K, Toda H, Wada M (2021). Colorectal Cancer Patients Have Four Specific Bacterial Species in Oral and Gut Microbiota in Common-A Metagenomic Comparison with Healthy Subjects. Cancers (Basel).

[27] Guven DC, Ergunay K, Brinkmann A, Alp A, Kittana FN, Akyon Y (2021). A Snapshot of Oral Microbiota in Patients with Colorectal Cancer. EJMI.

[28] Yang Y, Cai Q, Shu XO, Steinwandel MD, Blot WJ, Zheng W (2019). Prospective study of oral microbiome and colorectal cancer risk in low-income and African American populations. Int J Cancer.

[29] Komiya Y, Shimomura Y, Higurashi T, Sugi Y, Arimoto J, Umezawa S (2019). Patients with colorectal cancer have identical strains of Fusobacterium nucleatum in their colorectal cancer and oral cavity. Gut.

[30] Abed J, Maalouf N, Manson AL, Earl AM, Parhi L, Emgard JEM (2020). Colon Cancer-Associated Fusobacterium nucleatum May Originate From the Oral Cavity and Reach Colon Tumors via the Circulatory System. Front Cell Infect Microbiol.

[31] Chen F, Dai X, Zhou C-C, Li K-x, Zhang Y-j, Lou X-Y, et al. Integrated analysis of the faecal metagenome and serum metabolome reveals the role of gut microbiome-associated metabolites in the detection of colorectal cancer and adenoma. Gut. 2021:gutjnl-2020–323476. 10.1136/gutjnl-2020-323476 PMC9185821 34462336

[32] Kwong TNY, Wang X, Nakatsu G, Chow TC, Tipoe T, Dai RZW (2018). Association Between Bacteremia From Specific Microbes and Subsequent Diagnosis of Colorectal Cancer. Gastroenterology..

[33] Wang HF, Li LF, Guo SH, Zeng QY, Ning F, Liu WL (2016). Evaluation of antibody level against Fusobacterium nucleatum in the serological diagnosis of colorectal cancer. Sci Rep.

[34] Messaritakis I, Vogiatzoglou K, Tsantaki K, Ntretaki A, Sfakianaki M, Koulouridi A (2020). The Prognostic Value of the Detection of Microbial Translocation in the Blood of Colorectal Cancer Patients. Cancers (Basel).

[35] Xiao Q, Lu W, Kong X, Shao YW, Hu Y, Wang A (2021). Alterations of circulating bacterial DNA in colorectal cancer and adenoma: A proof-of-concept study. Cancer Lett.

[36] Butt J, Jenab M, Werner J, Fedirko V, Weiderpass E, Dahm CC (2021). Association of Pre-diagnostic Antibody Responses to Escherichia coli and Bacteroides fragilis Toxin Proteins with Colorectal Cancer in a European Cohort. Gut Microbes.

[37] Epplein M, Le Marchand L, Cover TL, Song M, Blot WJ, Peek RM (2020). Association of Combined Sero-Positivity to Helicobacter pylori and Streptococcus gallolyticus with Risk of Colorectal Cancer. Microorganisms.

[38] Butt J, Varga MG, Blot WJ, Teras L, Visvanathan K, Le Marchand L (2019). Serologic Response to Helicobacter pylori Proteins Associated With Risk of Colorectal Cancer Among Diverse Populations in the United States. Gastroenterology..

[39] Butt J, Romero-Hernandez B, Perez-Gomez B, Willhauck-Fleckenstein M, Holzinger D, Martin V (2016). Association of Streptococcus gallolyticus subspecies gallolyticus with colorectal cancer: Serological evidence. Int J Cancer.

[40] Butt J, Blot WJ, Teras LR, Visvanathan K, Le Marchand L, Haiman CA (2018). Antibody Responses to Streptococcus Gallolyticus Subspecies Gallolyticus Proteins in a Large Prospective Colorectal Cancer Cohort Consortium. Cancer Epidemiol Biomarkers Prev.

[41] Butt J, Jenab M, Willhauck-Fleckenstein M, Michel A, Pawlita M, Kyro C (2018). Prospective evaluation of antibody response to Streptococcus gallolyticus and risk of colorectal cancer. Int J Cancer.

[42] Taylor JC, Gao X, Xu J, Holder M, Petrosino J, Kumar R (2021). A type VII secretion system of Streptococcus gallolyticus subsp. gallolyticus contributes to gut colonization and the development of colon tumors. PLoS Pathog..

[43] Liu J, Ren L, Li S, Li W, Zheng X, Yang Y (2021). The biology, function, and applications of exosomes in cancer. Acta Pharm Sin B.

[44] Jia S, Zhang R, Li Z, Li J (2017). Clinical and biological significance of circulating tumor cells, circulating tumor DNA, and exosomes as biomarkers in colorectal cancer. Oncotarget.

[45] Cai X, Janku F, Zhan Q, Fan JB (2015). Accessing Genetic Information with Liquid Biopsies. Trends Genet.

[46] Siveen KS, Raza A, Ahmed EI, Khan AQ, Prabhu KS, Kuttikrishnan S (2019). The Role of Extracellular Vesicles as Modulators of the Tumor Microenvironment, Metastasis and Drug Resistance in Colorectal Cancer. Cancers (Basel).

[47] Halvaei S, Daryani S, Eslami SZ, Samadi T, Jafarbeik-Iravani N, Bakhshayesh TO (2018). Exosomes in Cancer Liquid Biopsy: A Focus on Breast Cancer. Mol Ther Nucleic Acids.

[48] Eylem CC, Yilmaz M, Derkus B, Nemutlu E, Camci CB, Yilmaz E (2020). Untargeted multi-omic analysis of colorectal cancer-specific exosomes reveals joint pathways of colorectal cancer in both clinical samples and cell culture. Cancer Lett.

[49] Chen Y, Xie Y, Xu L, Zhan S, Xiao Y, Gao Y (2017). Protein content and functional characteristics of serum-purified exosomes from patients with colorectal cancer revealed by quantitative proteomics. Int J Cancer.

[50] Ganig N, Baenke F, Thepkaysone ML, Lin K, Rao VS, Wong FC (2021). Proteomic Analyses of Fibroblast- and Serum-Derived Exosomes Identify QSOX1 as a Marker for Non-invasive Detection of Colorectal Cancer. Cancers (Basel).

[51] Li J, Chen Y, Guo X, Zhou L, Jia Z, Peng Z (2017). GPC1 exosome and its regulatory miRNAs are specific markers for the detection and target therapy of colorectal cancer. J Cell Mol Med.

[52] Sun B, Li Y, Zhou Y, Ng TK, Zhao C, Gan Q (2019). Circulating exosomal CPNE3 as a diagnostic and prognostic biomarker for colorectal cancer. J Cell Physiol.

[53] Lai PS, Chang WM, Chen YY, Lin YF, Liao HF, Chen CY (2021). Circulating microRNA-762 upregulation in colorectal cancer may be accompanied by Wnt-1/beta-catenin signaling. Cancer Biomark.

[54] Radwan E, Shaltout AS, Mansor SG, Shafik EA, Abbas WA, Shehata MR (2021). Evaluation of circulating microRNAs-211 and 25 as diagnostic biomarkers of colorectal cancer. Mol Biol Rep.

[55] Silva CMS, Barros-Filho MC, Wong DVT, Mello JBH, Nobre LMS, Wanderley CWS (2021). Circulating let-7e-5p, miR-106a-5p, miR-28–3p, and miR-542–5p as a Promising microRNA Signature for the Detection of Colorectal Cancer. Cancers (Basel).

[56] Karimi N, Ali Hosseinpour Feizi M, Safaralizadeh R, Hashemzadeh S, Baradaran B, Shokouhi B (2019). Serum overexpression of miR-301a and miR-23a in patients with colorectal cancer. J Chin Med Assoc.

[57] Concepcion CP, Bonetti C, Ventura A (2012). The microRNA-17-92 family of microRNA clusters in development and disease. Cancer J.

[58] Matsumura T, Sugimachi K, Iinuma H, Takahashi Y, Kurashige J, Sawada G (2015). Exosomal microRNA in serum is a novel biomarker of recurrence in human colorectal cancer. Br J Cancer.

[59] Barbagallo C, Brex D, Caponnetto A, Cirnigliaro M, Scalia M, Magnano A (2018). LncRNA UCA1, Upregulated in CRC Biopsies and Downregulated in Serum Exosomes, Controls mRNA Expression by RNA-RNA Interactions. Mol Ther Nucleic Acids.

[60] Liu T, Zhang X, Gao S, Jing F, Yang Y, Du L (2016). Exosomal long noncoding RNA CRNDE-h as a novel serum-based biomarker for diagnosis and prognosis of colorectal cancer. Oncotarget.

[61] Wang M, Su Z, Amoah Barnie P (2020). Crosstalk among colon cancer-derived exosomes, fibroblast-derived exosomes, and macrophage phenotypes in colon cancer metastasis. Int Immunopharmacol..

[62] Zheng X, Liu J, Li X, Tian R, Shang K, Dong X (2021). Angiogenesis is promoted by exosomal DPP4 derived from 5-fluorouracil-resistant colon cancer cells. Cancer Lett.

[63] Takano Y, Masuda T, Iinuma H, Yamaguchi R, Sato K, Tobo T (2017). Circulating exosomal microRNA-203 is associated with metastasis possibly via inducing tumor-associated macrophages in colorectal cancer. Oncotarget.

[64] Teng Y, Ren Y, Hu X, Mu J, Samykutty A, Zhuang X (2017). MVP-mediated exosomal sorting of miR-193a promotes colon cancer progression. Nat Commun.

[65] Tsukamoto M, Iinuma H, Yagi T, Matsuda K, Hashiguchi Y (2017). Circulating Exosomal MicroRNA-21 as a Biomarker in Each Tumor Stage of Colorectal Cancer. Oncology.

[66] Fu F, Jiang W, Zhou L, Chen Z (2018). Circulating Exosomal miR-17-5p and miR-92a-3p Predict Pathologic Stage and Grade of Colorectal Cancer. Transl Oncol.

[67] Zeng Z, Li Y, Pan Y, Lan X, Song F, Sun J (2018). Cancer-derived exosomal miR-25-3p promotes pre-metastatic niche formation by inducing vascular permeability and angiogenesis. Nat Commun.

[68] Yaffee P, Osipov A, Tan C, Tuli R, Hendifar A (2015). Review of systemic therapies for locally advanced and metastatic rectal cancer. J Gastrointest Oncol.

[69] Hu JL, Wang W, Lan XL, Zeng ZC, Liang YS, Yan YR (2019). CAFs secreted exosomes promote metastasis and chemotherapy resistance by enhancing cell stemness and epithelial-mesenchymal transition in colorectal cancer. Mol Cancer.

[70] Huber V, Fais S, Iero M, Lugini L, Canese P, Squarcina P (2005). Human colorectal cancer cells induce T-cell death through release of proapoptotic microvesicles: role in immune escape. Gastroenterology.

[71] Liang G, Zhu Y, Ali DJ, Tian T, Xu H, Si K (2020). Engineered exosomes for targeted co-delivery of miR-21 inhibitor and chemotherapeutics to reverse drug resistance in colon cancer. J Nanobiotechnology.

[72] Yan S, Liu G, Jin C, Wang Z, Duan Q, Xu J (2018). MicroRNA-6869-5p acts as a tumor suppressor via targeting TLR4/NF-kappaB signaling pathway in colorectal cancer. J Cell Physiol.

[73] Ren D, Lin B, Zhang X, Peng Y, Ye Z, Ma Y (2017). Maintenance of cancer stemness by miR-196b-5p contributes to chemoresistance of colorectal cancer cells via activating STAT3 signaling pathway. Oncotarget.

[74] Hu YB, Yan C, Mu L, Mi YL, Zhao H, Hu H (2019). Exosomal Wnt-induced dedifferentiation of colorectal cancer cells contributes to chemotherapy resistance. Oncogene.

[75] Jin G, Liu Y, Zhang J, Bian Z, Yao S, Fei B (2019). A panel of serum exosomal microRNAs as predictive markers for chemoresistance in advanced colorectal cancer. Cancer Chemother Pharmacol.

[76] Liu T, Zhang X, Du L, Wang Y, Liu X, Tian H (2019). Exosome-transmitted miR-128-3p increase chemosensitivity of oxaliplatin-resistant colorectal cancer. Mol Cancer.

[77] Li Y, Gao Y, Gong C, Wang Z, Xia Q, Gu F (2018). A33 antibody-functionalized exosomes for targeted delivery of doxorubicin against colorectal cancer. Nanomedicine.

[78] Thomson T, Lin H (2009). The biogenesis and function of PIWI proteins and piRNAs: progress and prospect. Ann Rev Cell Dev.

[79] Ku HY, Lin H (2014). PIWI proteins and their interactors in piRNA biogenesis, germline development and gene expression. Natl Sci Rev.

[80] Sabbah NA, Abdalla WM, Mawla WA, AbdAlMonem N, Gharib AF, Abdul-Saboor A (2021). piRNA-823 Is a Unique Potential Diagnostic Non-Invasive Biomarker in Colorectal Cancer Patients. Genes.

[81] Wang Z, Yang H, Ma D, Mu Y, Tan X, Hao Q (2020). Serum PIWI-interacting RNAs piR-020619 and piR-020450 are promising novel biomarkers for early detection of colorectal cancer. Cancer Epidemiol Prev Biomark.

[82] Vychytilova-Faltejskova P, Stitkovcova K, Radova L, Sachlova M, Kosarova Z, Slaba K (2018). Circulating PIWI-interacting RNAs piR-5937 and piR-28876 are promising diagnostic biomarkers of colon cancer. Cancer Epidemiol Prev Biomark.

[83] Yin J, Jiang XY, Qi W, Ji CG, Xie XL, Zhang DX (2017). piR-823 contributes to colorectal tumorigenesis by enhancing the transcriptional activity of HSF 1. Cancer Sci.

[84] lyer DN, Wan TM-H, Man JH-W, Sin RW-Y, Li X, Lp OS-H (2020). Small RNA profiling of piRNAs in colorectal cancer identifies consistent overexpression of piR-24000 that correlates clinically with an aggressive disease phenotype. Cancers.

[85] Weng W, Liu N, Toiyama Y, Kusunoki M, Nagasaka T, Fujiwara T (2018). Novel evidence for a PIWI-interacting RNA (piRNA) as an oncogenic mediator of disease progression, and a potential prognostic biomarker in colorectal cancer. Mol Cancer.

[86] Qu A, Wang W, Yang Y, Zhang X, Dong Y, Zheng G (2019). A serum piRNA signature as promising non-invasive diagnostic and prognostic biomarkers for colorectal cancer. Cancer Manag Res.

[87] Mai D, Ding P, Tan L, Zhang J, Pan Z, Bai R (2018). PIWI-interacting RNA-54265 is oncogenic and a potential therapeutic target in colorectal adenocarcinoma. Theranostics.

[88] Mai D, Zheng Y, Guo H, Ding P, Bai R, Li M (2020). Serum piRNA-54265 is a New Biomarker for early detection and clinical surveillance of Human Colorectal Cancer. Theranostics.

[89] Shen X, Xue Y, Cong H, Wang X, Fan Z, Cui X, et al. Circulating lncRNA DANCR as a potential auxillary biomarker for the diagnosis and prognostic prediction of colorectal cancer. Biosci Rep. 2020;40(3). 10.1042/BSR20191481 PMC7103578 32159208

[90] Abedini P, Fattahi A, Agah S, Talebi A, Beygi AH, Amini SM (2019). Expression analysis of circulating plasma long noncoding RNAs in colorectal cancer: The relevance of lncRNAs ATB and CCAT1 as potential clinical hallmarks. J Cell Physiol.

[91] Ding Y, Feng W, Ge J-K, Dai L, Liu T-t, Hua X-y (2020). Serum level of long noncoding RNA B3GALT5-AS1 as a diagnostic biomarker of colorectal cancer. Future Oncology.

[92] Wang W, Xie Y, Chen F, Liu X, Zhong L-L, Wang H-Q (2019). LncRNA MEG3 acts a biomarker and regulates cell functions by targeting ADAR1 in colorectal cancer. World J Gastroenterol.

[93] Xu W, Zhou G, Wang H, Liu Y, Chen B, Chen W (2020). Circulating lncRNA SNHG11 as a novel biomarker for early diagnosis and prognosis of colorectal cancer. Int J Cancer.

[94] Samir N, Matboli M, El-Tayeb H, El-Tawdi A, Hassan MK, Waly A (2018). Competing endogenous RNA network crosstalk reveals novel molecular markers in colorectal cancer. J Cell Biochem.

[95] Li J, Song Y, Wang J, Huang J (2020). Plasma circular RNA panel acts as a novel diagnostic biomarker for colorectal cancer detection. Am J Transl Res.

[96] Lin J, Cai D, Li W, Yu T, Mao H, Jiang S (2019). Plasma circular RNA panel acts as a novel diagnostic biomarker for colorectal cancer. Clin Biochem.

[97] Li XN, Wang ZJ, Ye CX, Zhao BC, Huang XX, Yang L (2019). Circular RNA circVAPA is up-regulated and exerts oncogenic properties by sponging miR-101 in colorectal cancer. Biomed Pharmacother..

[98] Ye DX, Wang SS, Huang Y, Chi P (2019). A 3-circular RNA signature as a noninvasive biomarker for diagnosis of colorectal cancer. Cancer Cell Int.

[99] Xing L, Xia M, Jiao X, Fan L (2020). Hsa_circ_0004831 serves as a blood-based prognostic biomarker for colorectal cancer and its potentially circRNA-miRNA-mRNA regulatory network construction. Cancer Cell Int.

[100] Zhang W, Yang S, Liu Y, Wang Y, Lin T, Li Y (2018). Hsa_circ_0007534 as a blood-based marker for the diagnosis of colorectal cancer and its prognostic value. Int J Clin Exp Pathol.

[101] Ji W, Qiu C, Wang M, Mao N, Wu S, Dai Y (2018). Hsa_circ_0001649: A circular RNA and potential novel biomarker for colorectal cancer. Biochem Biophys Res Commun.

[102] Yang N, Xu B, Kong P, Han M, Li BH (2020). Hsa_circ_0002320: a novel clinical biomarker for colorectal cancer prognosis. Medicine (Baltimore)..

[103] Mai S, Zhang Z, Mi W (2021). Upregulation of circ_PVT1 and circ_001569 Indicate Unfavorable Prognosis in Colorectal Cancer. Ann Clin Lab Sci.

[104] Tian J, Xi X, Wang J, Yu J, Huang Q, Ma R (2019). CircRNA hsa_circ_0004585 as a potential biomarker for colorectal cancer. Cancer Manag Res.

[105] Sazanov AA, Kiselyova EV, Zakharenko AA, Romanov MN, Zaraysky MI (2017). Plasma and saliva miR-21 expression in colorectal cancer patients. J Appl Genet.

[106] Gmerek L, Martyniak K, Horbacka K, Krokowicz P, Scierski W, Golusinski P (2019). MicroRNA regulation in colorectal cancer tissue and serum. PLoS One..

[107] Yamada NO, Senda T (2021). Circulating microRNA-92a-3p in colorectal cancer: a review. Med Mol Morphol.

[108] Radanova M, Mihaylova G, Mihaylova Z, Ivanova D, Tasinov O, Nazifova-Tasinova N (2021). Circulating miR-618 Has Prognostic Significance in Patients with Metastatic Colon Cancer. Curr Oncol.

[109] Nassar FJ, Msheik ZS, Itani MM, Helou RE, Hadla R, Kreidieh F, et al. Circulating miRNA as Biomarkers for Colorectal Cancer Diagnosis and Liver Metastasis. Diagnostics (Basel). 2021;11(2). 10.3390/diagnostics11020341 PMC7921943 33669508

[110] Fu D, Chen Y, Xu D (2021). Circulating miR-449a predicts survival outcome for colorectal cancer following curative resection: An observational study. Medicine (Baltimore)..

[111] Abdul-Maksoud RS, Elsayed RS, Elsayed WSH, Sediq AM, Rashad NM, Shaker SE (2021). Combined serum miR-29c and miR-149 expression analysis as diagnostic genetic markers for colorectal cancer. Biotechnol Appl Biochem.

[112] Bader El Din NG, Farouk S, Abdel-Salam LO, Khairy A. The potential value of miRNA-223 as a diagnostic biomarker for Egyptian colorectal patients. Eur J Gastroenterol Hepatol. 2021;33(1):25–31. 10.1097/MEG.0000000000001961 33079781

[113] Dokhanchi M, Pakravan K, Zareian S, Hussen BM, Farid M, Razmara E (2021). Colorectal cancer cell-derived extracellular vesicles transfer miR-221–3p to promote endothelial cell angiogenesis via targeting suppressor of cytokine signaling 3. Life Sci..

[114] Peng X, Wang J, Zhang C, Liu K, Zhao L, Chen X (2020). A three-miRNA panel in serum as a noninvasive biomarker for colorectal cancer detection. Int J Biol Markers.

[115] Zhang Z, Zhang D, Cui Y, Qiu Y, Miao C, Lu X (2020). Identification of microRNA-451a as a Novel Circulating Biomarker for Colorectal Cancer Diagnosis. Biomed Res Int.

[116] Farouk S, Khairy A, Salem AM, Soliman AF, Bader El Din NG (2020). Differential Expression of miR-21, miR-23a, and miR-27a, and Their Diagnostic Significance in Egyptian Colorectal Cancer Patients. Genet Test Mol Biomarkers.

[117] Gasparello J, Papi C, Allegretti M, Giordani E, Carboni F, Zazza S, et al. A Distinctive microRNA (miRNA) Signature in the Blood of Colorectal Cancer (CRC) Patients at Surgery. Cancers (Basel). 2020;12(9):2410. 10.3390/cancers12092410. 10.3390/cancers12092410 PMC7564483 32854257

[118] Shaker OG, Ayeldeen G, Abdelhamid AM. Circulating microRNA-944 and its target gene EPHA7 as a potential biomarker for colorectal cancer. Arch Physiol Biochem. 2020:1–7. 10.1080/13813455.2020.1762658 32421395

[119] Ahmed Hassan E, El-Din Abd El-Rehim AS, Mohammed Kholef EF, Abd-Elgwad Elsewify W (2020). Potential role of plasma miR-21 and miR-92a in distinguishing between irritable bowel syndrome, ulcerative colitis, and colorectal cancer. Gastroenterol Hepatol Bed Bench.

[120] Pan Z, Miao L (2020). Serum microRNA-592 serves as a novel potential biomarker for early diagnosis of colorectal cancer. Oncol Lett.

[121] Sun Y, An K, Yang C. Circulating Cell-Free DNA, Liquid Biopsy. In: Strumfa I, Gardovskis J, editors. IntechOpen. 2019. 10.5772/intechopen.80730.

[122] Li G, Pavlick D, Chung JH, Bauer T, Tan BA, Peguero J (2019). Genomic profiling of cell-free circulating tumor DNA in patients with colorectal cancer and its fidelity to the genomics of the tumor biopsy. J Gastrointest Oncol.

[123] Toor OM, Ahmed Z, Bahaj W, Boda U, Cummings LS, McNally ME (2018). Correlation of Somatic Genomic Alterations Between Tissue Genomics and ctDNA Employing Next-Generation Sequencing: Analysis of Lung and Gastrointestinal Cancers. Mol Cancer Ther.

[124] Kasi PM, Kamatham S, Shahjehan F, Li Z, Johnson PW, Merchea A, Colibaseanu DT. Liquid biopsy concordance based on clonality and timing of testing in patients with metastatic colorectal cancer. Ann Oncol. 2019.

[125] Alcaide M, Cheung M, Hillman J, Rassekh SR, Deyell RJ, Batist G (2020). Evaluating the quantity, quality and size distribution of cell-free DNA by multiplex droplet digital PCR. Sci Rep.

[126] Flamini E, Mercatali L, Nanni O, Calistri D, Nunziatini R, Zoli W (2006). Free DNA and carcinoembryonic antigen serum levels: an important combination for diagnosis of colorectal cancer. Clin Cancer Res.

[127] Boni L, Cassinotti E, Canziani M, Dionigi G, Rovera F, Dionigi R (2007). Free circulating DNA as possible tumour marker in colorectal cancer. Surg Oncol.

[128] Frattini M, Gallino G, Signoroni S, Balestra D, Battaglia L, Sozzi G (2006). Quantitative analysis of plasma DNA in colorectal cancer patients: a novel prognostic tool. Ann N Y Acad Sci.

[129] Spindler KL, Pallisgaard N, Andersen RF, Brandslund I, Jakobsen A (2015). Circulating free DNA as biomarker and source for mutation detection in metastatic colorectal cancer. PloS one..

[130] Yeh YM, Lin PC, Lee CT, Chen SH, Lin BW, Lin SC (2020). Treatment monitoring of colorectal cancer by integrated analysis of plasma concentration and sequencing of circulating tumor DNA. Mol Cancer.

[131] Corcoran RB, Chabner BA (2018). Application of Cell-free DNA Analysis to Cancer Treatment. N Engl J Med.

[132] Cristiano S, Leal A, Phallen J, Fiksel J, Adleff V, Bruhm DC (2019). Genome-wide cell-free DNA fragmentation in patients with cancer. Nature.

[133] Kerachian MA, Javadmanesh A, Azghandi M, Mojtabanezhad Shariatpanahi A, Yassi M, Shams Davodly E (2020). Crosstalk between DNA methylation and gene expression in colorectal cancer, a potential plasma biomarker for tracing this tumor. Sci Rep.

[134] Laugsand EA, Brenne SS, Skorpen F (2021). DNA methylation markers detected in blood, stool, urine, and tissue in colorectal cancer: a systematic review of paired samples. Int J Colorectal Dis.

[135] Young GP, Symonds EL, Nielsen HJ, Ferm L, Christensen IJ, Dekker E (2021). Evaluation of a panel of tumor-specific differentially-methylated DNA regions in IRF4, IKZF1 and BCAT1 for blood-based detection of colorectal cancer. Clin Epigenetics.

[136] Wang D, O'Rourke D, Sanchez-Garcia JF, Cai T, Scheuenpflug J, Feng Z (2021). Development of a liquid biopsy based purely quantitative digital droplet PCR assay for detection of MLH1 promoter methylation in colorectal cancer patients. BMC Cancer.

[137] Sui J, Wu X, Wang C, Wang G, Li C, Zhao J (2021). Discovery and validation of methylation signatures in blood-based circulating tumor cell-free DNA in early detection of colorectal carcinoma: a case-control study. Clin Epigenetics.

[138] Li D, Zhang L, Fu J, Huang H, Sun S, Zhang D (2020). SCTR hypermethylation is a diagnostic biomarker in colorectal cancer. Cancer Sci.

[139] Chang SC, Liew PL, Ansar M, Lin SY, Wang SC, Hung CS (2020). Hypermethylation and decreased expression of TMEM240 are potential early-onset biomarkers for colorectal cancer detection, poor prognosis, and early recurrence prediction. Clin Epigenetics.

[140] Li J, Zhou X, Liu X, Ren J, Wang J, Wang W (2019). Detection of Colorectal Cancer in Circulating Cell-Free DNA by Methylated CpG Tandem Amplification and Sequencing. Clin Chem.

[141] Wu X, Zhang Y, Hu T, He X, Zou Y, Deng Q, et al. A novel cell-free DNA methylation-based model improves the early detection of colorectal cancer. Mol Oncol. 2021. 10.1002/1878-0261.12942 PMC8486566 33694305

[142] Jensen SO, Ogaard N, Orntoft MW, Rasmussen MH, Bramsen JB, Kristensen H (2019). Novel DNA methylation biomarkers show high sensitivity and specificity for blood-based detection of colorectal cancer-a clinical biomarker discovery and validation study. Clin Epigenetics.

[143] Tie J, Cohen JD, Lo SN, Wang Y, Li L, Christie M (2021). Prognostic significance of postsurgery circulating tumor DNA in nonmetastatic colorectal cancer: Individual patient pooled analysis of three cohort studies. Int J Cancer.

[144] Osumi H, Shinozaki E, Yamaguchi K, Zembutsu H (2019). Early change in circulating tumor DNA as a potential predictor of response to chemotherapy in patients with metastatic colorectal cancer. Sci Rep.

[145] van Helden EJ, Angus L, Menke-van der Houven van Oordt CW, Heideman DAM, Boon E, van Es SC (2019). RAS and BRAF mutations in cell-free DNA are predictive for outcome of cetuximab monotherapy in patients with tissue-tested RAS wild-type advanced colorectal cancer. Mol Oncol.

[146] Yamada T, Matsuda A, Takahashi G, Iwai T, Takeda K, Ueda K (2020). Emerging RAS, BRAF, and EGFR mutations in cell-free DNA of metastatic colorectal patients are associated with both primary and secondary resistance to first-line anti-EGFR therapy. Int J Clin Oncol.

[147] van 't Erve I, Greuter MJE, Bolhuis k, Vessies DCL, Leal A, Vink GR (2020). Diagnostic Strategies toward Clinical Implementation of Liquid Biopsy RAS/BRAF Circulating Tumor DNA Analyses in Patients with Metastatic Colorectal Cancer. J Mol Diagn.

[148] Wang C, Chevalier D, Saluja J, Sandhu J, Lau C, Fakih M (2020). Regorafenib and Nivolumab or Pembrolizumab Combination and Circulating Tumor DNA Response Assessment in Refractory Microsatellite Stable Colorectal Cancer. Oncologist.

[149] Kasi P, Chan C (2020). 23 Circulating tumor DNA (ctDNA) serial analysis during progression on PD-1 blockade and later CTLA4 rescue in patients with mismatch repair deficient metastatic colorectal cancer. J Immunother Cancer.

[150] Pantel K, Alix-Panabieres C (2010). Circulating tumour cells in cancer patients: challenges and perspectives. Trends Mol Med.

[151] Allard WJ, Matera J, Miller MC, Repollet M, Connelly MC, Rao C (2004). Tumor cells circulate in the peripheral blood of all major carcinomas but not in healthy subjects or patients with nonmalignant diseases. Clin Cancer Res.

[152] Kalikaki A, Politaki H, Souglakos J, Apostolaki S, Papadimitraki E, Georgoulia N (2014). KRAS genotypic changes of circulating tumor cells during treatment of patients with metastatic colorectal cancer. PLoS One..

[153] Konczalla L, Wostemeier A, Kemper M, Karstens KF, Izbicki J, Reeh M. Clinical Significance of Circulating Tumor Cells in Gastrointestinal Carcinomas. Diagnostics (Basel). 2020;10(4). 10.3390/diagnostics10040192 PMC7235836 32235479

[154] Delgado-Urena M, Ortega FG, de Miguel-Perez D, Rodriguez-Martinez A, Garcia-Puche JL, Ilyine H (2018). Circulating tumor cells criteria (CyCAR) versus standard RECIST criteria for treatment response assessment in metastatic colorectal cancer patients. J Transl Med.

[155] Yang C, Zhuang W, Hu Y, Zhu L (2018). Clinical significance of peripheral circulating tumor cell counts in colorectal polyps and non-metastatic colorectal cancer. World J Surg Oncol.

[156] Tsai WS, You JF, Hung HY, Hsieh PS, Hsieh B, Lenz HJ (2019). Novel Circulating Tumor Cell Assay for Detection of Colorectal Adenomas and Cancer. Clin Transl Gastroenterol..

[157] Pan RJ, Hong HJ, Sun J, Yu CR, Liu HS, Li PY (2021). Detection and Clinical Value of Circulating Tumor Cells as an Assisted Prognostic Marker in Colorectal Cancer Patients. Cancer Manag Res.

[158] Bidard FC, Kiavue N, Ychou M, Cabel L, Stern MH, Madic J, et al. Circulating Tumor Cells and Circulating Tumor DNA Detection in Potentially Resectable Metastatic Colorectal Cancer: A Prospective Ancillary Study to the Unicancer Prodige-14 Trial. Cells. 2019;8(6). 10.3390/cells8060516 PMC6627974 31142037

[159] Camera S, Akin Telli T, Woff E, Vandeputte C, Kehagias P, Guiot T, et al. Prognostic Value of the Pace of Tumor Progression as Assessed by Serial (18)F-FDG PET/CT Scan and Liquid Biopsy in Refractory Colorectal Cancer: The CORIOLAN Trial. Cancers (Basel). 2020;12(10). 10.3390/cancers12102752 PMC7601470 32987838

[160] Aranda E, Vieitez JM, Gomez-Espana A, Gil Calle S, Salud-Salvia A, Grana B (2020). FOLFOXIRI plus bevacizumab versus FOLFOX plus bevacizumab for patients with metastatic colorectal cancer and >/=3 circulating tumour cells: the randomised phase III VISNU-1 trial. ESMO Open..

[161] Troncarelli Flores BC, Souza ESV, Ali Abdallah E, Mello CAL, Gobo Silva ML, Gomes Mendes G, et al. Molecular and Kinetic Analyses of Circulating Tumor Cells as Predictive Markers of Treatment Response in Locally Advanced Rectal Cancer Patients. Cells. 2019;8(7). 10.3390/cells8070641 PMC6679115 31247977

[162] Shou X, Li Y, Hu W, Ye T, Wang G, Xu F (2019). Six-gene Assay as a new biomarker in the blood of patients with colorectal cancer: establishment and clinical validation. Mol Oncol.

